# A predominance of R5-like HIV genotypes in vaginal secretions is associated with elevated plasma HIV-1 RNA levels and the absence of anti-retroviral therapy

**DOI:** 10.1186/1743-422X-5-87

**Published:** 2008-07-29

**Authors:** Tara C Randolph, Patricia J Kissinger, Rebecca A Clark, Nedra Lacour, Angela M Amedee

**Affiliations:** 1 Department of Microbiology, Immunology, and Parasitology, Louisiana State University Health Sciences Center, New Orleans, LA, USA; 2 School of Public Health and Tropical Medicine, Tulane University Health Sciences Center, New Orleans, LA, USA

## Abstract

HIV expressed in genital secretions provides the inoculum from which transmitting variants are selected, both in sexual transmission and mother-to-infant transmission during partuition. Characterization of HIV levels and genotypes found in vaginal secretions and the impact of anti-retroviral therapy (ART) on this virus can provide valuable insight for the prevention of HIV transmission. Vaginal HIV was evaluated in a cohort of 43 women attending a New Orleans HIV outpatient clinic. Predominant vaginal genotypes were characterized as R5- or X4-like by heteroduplex tracking analyses of the envelope V3 region. Most women (67.4%) shed R5-like genotypes in vaginal secretions which was associated with elevated plasma HIV levels (≥ 10,000 copies HIV-RNA/mL) and absence of ART. Because R5-like genotypes are more frequently associated with transmission, these observations suggest that the majority of women shedding HIV in genital secretions present a transmission risk. The levels of vaginal virus were similar between both groups, but shedding of X4-like genotypes was associated with lower plasma viral loads and the use of ART, suggesting that ART use may impact the genotypes of virus found in the female genital compartment.

## Findings

Approximately half of the world's HIV-infected population is female, and the rate of infection is increasing in this population [1]. Mother-to-infant transmission of HIV continues to be a dire issue in endemic areas such as Sub-Saharan Africa and the Caribbean [1,2]. Currently, heterosexual transmission is the primary route of HIV infection [1], and studies of discordant couples in Sub-Saharan Africa highlight a growing problem of female to male sexual transmission events [3,4]. Because virus contained in vaginal secretions likely comprises the inoculum from which transmitting variants are selected in mother-to-infant transmission during partuition, as well as through sexual contact, identifying the specific properties and genotypes of virus expressed in this compartment will aid in the design of strategies to prevent HIV transmission.

Studies have described the compartmentalized replication of vaginal HIV as compared to plasma [5,6]. Viral tropism and the ability of virions to infect cell types that may traffic to specific tissues, such as the vaginal mucosa, are influenced by the virus' envelope sequence, which determines the molecules utilized for entry [7]. The nucleotide sequence of the HIV envelope V3 region determines whether the virion utilizes a CCR5 (R5) or CXCR4 (X4) coreceptor molecule, therefore the V3 genotype can impact compartmentalized replication of virus in the genital mucosa [5,7,8].

To evaluate the properties of HIV expressed in the genital tract and the potential impact of ART on genital tract viral genotypes, we measured HIV-1 RNA levels in vaginal secretions and plasma, determined the V3 genotype of predominant variants found in vaginal secretions, and assessed ART use in a cohort of 43 women attending an HIV outpatient clinic in New Orleans. Informed consent was obtained in accordance with Louisiana State University Health Sciences Center and Tulane Medical Center IRB approval. ART use was evaluated by the subject's answer to the question "Are you currently taking ART?" on a survey questionnaire completed at the study visit. ART use was also confirmed by review of medical chart data. Vaginal secretions were collected from the vaginal vault on Dacron swabs and subsequently eluted in phosphate-buffered solution. Supernatant and cell fractions were stored at -80°C within three hours of collection. Samples were not collected during menses, and overtly blood-contaminated samples were excluded. Blood samples were collected in ethylenediaminetetraacetic acid (EDTA) anti-coagulant tubes, and plasma aliquots were stored frozen at -80°C. HIV contained in plasma and vaginal secretion supernatant samples was collected by centrifugation and virion-associated HIV-1 RNA copies were quantified using the Roche Amplicor™ Ultra-Sensitive HIV-1 Monitor Assay, according to assay protocol.

Samples from a cohort of 43 women with measurable levels (>50 copies/sample) of vaginal HIV-1 RNA were available for genotypic analysis in this study. To evaluate viral genotypes shed in vaginal secretions, the envelope V3 region was amplified by nested RT-PCR from the remaining isolated RNA and analyzed by a heteroduplex tracking assay (HTA) based on an established protocol [9]. This methodology provides a facile means to evaluate nucleotide changes between a reference sequence and sequences amplified from each vaginal sample. This protocol has been utilized and validated for analyses of HIV genotypes and prediction of coreceptor usage [9-13]. The genotypic evaluations by heteroduplex analysis in this study were conducted using sample-derived HIV-V3 PCR products, pooled from 2–4 independent PCR amplications. The 334 base pair V3 PCR products were mixed with a single-stranded ^32^P-radiolabeled probe containing the V3 sequence of an X4-utilizing strain, HIV_HXB_. Following electrophoresis on a non-denaturing acrylamide gel, mobility ratios of the heteroduplexes formed between sample and probe V3 sequences were analyzed and used to determine the predominant genotype of the patient sample, as described [9,12,13]. R5-like genotypes had a reduced mobility on the gel when heteroduplexed with the X4-like sequence probe (mobility ratio < 0.91), while heteroduplexes formed between patient X4-like sequences and the X4 probe had higher mobility ratios (>0.91).

A wide range of viral levels in both the plasma and vaginal compartments was observed in this cohort, 50 to 5 × 10^5 ^copies/mL plasma and 50 to 2 × 10^5 ^copies/vaginal sample. For statistical analysis, HIV RNA levels in both plasma and vaginal secretions were categorically designated as high-level HIV or low-level HIV for each, as defined in Table 1. The majority of women, 67.4%, had R5-like genotypes in vaginal secretions, while 32.6% shed X4-like genotypes. To identify viral and clinical factors associated with vaginal X4- or R5-like genotypes, we evaluated plasma and vaginal viral levels, ART use, and CD4+ T lymphoctye counts using Chi-square and Fisher's exact tests (Table 1). R5-like vaginal HIV genotypes were associated with high levels of plasma virus and the absence of ART. Conversely, X4-like genotypes in vaginal fluids correlated with lower plasma levels and use of ART. Levels of HIV in vaginal secretions were similar between both groups.

**Table 1:** Vaginal Genotypic Associations in 43 Women

	**R5-like Genotypes** ** (n = 29)**	**X4-like Genotypes ** **(n = 14)**	*p*-value*
High Plasma RNA Levels ≥ 10,000 copies HIV RNA/mL	62.1%	14.3%	**0.01**
Low Plasma RNA Levels < 1,000 copies HIV RNA/mL	20.7%	71.4%	**0.004**
High Vaginal RNA Levels > 500 copies HIV RNA/sample	48.3%	44.4%	1.00
Low Vaginal RNA Levels < 100 copies HIV RNA/sample	41.4%	42.9%	0.81
ART Use	38.0%	85.7%	**0.01**
< 200 CD4+ T lymphocytes/ul	34.6%	44%	0.44^#^

*Chi Square (*Χ*^2^) analyses

#Fisher Exact Test; data from 81% of the cohort available for analysis

The presence of predominantly R5-like genotypes in the vaginal compartment has significant implications for HIV transmission. Genotypes that utilize the CCR5 coreceptor are most commonly transmitted in both sexual and mother-to-infant transmission [14]. This collective data suggests the majority of women shedding vaginal HIV present a high transmission risk. Furthermore, vaginal R5-like genotypes were associated with absence of ART use. Reasons for ART use/non-use were not documented for this cohort and are often multifactorial, especially in women. Subjects taking ART (n = 23) and those not taking ART (n = 20) had similar levels of peripheral CD4+ T lymphocytes, indicating that advanced disease was not the main factor driving ART use in this cohort. Additionally, subjects shedding R5-like genotypes as compared to women with X4-like vaginal genotypes had similar levels of peripheral CD4+ T lymphocytes (Table 1). Plasma levels of HIV were lower in ART users as compared to non-users, however ART users represented more than half of the women in this shedding cohort, demonstrating that vaginal HIV shedding occurs in a number of women taking ART. ART use was associated with the presence of predominantly X4-like variants in vaginal secretions, which are less likely to be transmitted [14]. These observations suggest that ART effectively controls the expression of R5-like genotypes in the vaginal compartment.

Due to sample availability, HTA analyses were not conducted on matched plasma samples. Although it is possible to harbor dual-tropic viral variants [9,13], the low levels of HIV detected in many of the samples only allowed the analysis of the predominant genotypes in genital secretions. Despite this, and the inherent limitations of PCR amplification methodologies, these preliminary findings demonstrate ART's impact on genital HIV genotypes. These data provide a basis for future evaluations of HIV treatment in women to fully examine compartmentalized vaginal expression and the effectiveness of ART on X4-like and R5-like genotypes in vaginal secretions as a means to reduce transmission risk.

## List of Abbreviations

HIV: Human immunodeficiency virus; ART: Antiretroviral therapy; CCR5: Chemokine (C-C motif) receptor 5; CXCR4: Chemokine (C-X-C motif) receptor 4; RNA: Ribonucleic acid; PCR: Polymerase chain reaction; IRB: Institutional review board.

## Competing interests

The authors declare that they have no competing interests.

## Authors' contributions

TR conducted HTA analyses, participated in PCR sample preparation, carried out data analysis, and drafted the manuscript. PK participated in the study design and conducted the statistical analyses. RC participated in the design and coordination of the study and facilitated the collection of clinical samples. NL processed clinical samples and conducted PCR and viral load analyses. AA participated in the coordination of the study, directed sample and data analysis, and assisted in drafting the manuscript.
